# Citric acid impairs type B trichothecene biosynthesis of *Fusarium graminearum* but enhances its growth and pigment biosynthesis: transcriptomic and proteomic analyses

**DOI:** 10.1128/aem.01531-24

**Published:** 2025-05-14

**Authors:** Luzhou Cai, Ling Li, Dong Li, Yanping Wu, Jinrong Bai, Kai Zhong, Hong Gao

**Affiliations:** 1 College of Biomass Science and Engineering, Sichuan University 546365, Chengdu, China; 2 College of Bioengineering, Sichuan University of Science and Engineering, Yibin, China; 3 Molecular Toxicology Key Laboratory of Sichuan Provincial Education Office, West China School of Public Health, West China Fourth Hospital, Sichuan University 12530, Chengdu, China; The University of Tennessee Knoxville, Knoxville, Tennessee, USA

**Keywords:** citric acid, *F. graminearum*, trichothecene, transcriptomic, proteomic

## Abstract

*Fusarium graminearum* is a pathogenic fungus that causes devastating plant diseases such as Fusarium head blight, leading to significant food waste and posing a threat to human health due to the accumulation of mycotoxins. As a soil-borne fungus, *F. graminearum* interacts closely with plants and soil, completing its life cycle in an intricate environment. In this investigation, citric acid (CA), a plant root exudate and heavy metal chelator, was investigated for its effect on *F. graminearum* at concentrations of 2.5, 5, 10, and 20 mM. The fungus treated with this CA gradient exhibited different growth conditions and mycelial colors. Integrative analysis of transcriptomics and proteomics found that the growth of the fungus was accelerated by the upregulation of carbon source metabolism-related enzymes such as phosphoenolpyruvate carboxykinase (PEPCK), Glpk, and LAI12, and the mycelial pigments were altered by polyketide biosynthetic enzymes like AurF, AurJ, and AurT, which were responsible for the production of aurofusarin and rubrofusarin. Interestingly, many *Tri* genes and their corresponding proteins along with type B trichothecene biosynthesis were significantly downregulated, though the fungal growth was promoted. In this study, the CK, 2.5, and 5 mM groups had similar expression patterns of RNA and protein, while the 10 and 20 mM groups were alike. With the validation of CK, 5, and 10 mM groups, the discovery of CA as a type B trichothecene biosynthesis inhibitor and growth promoter was expected to facilitate its reasonable application and contribute to further research for the prevention of *F. graminearum*.

**IMPORTANCE:**

*Fusarium graminearum* is a challenging phytopathogen that causes plant disasters worldwide, such as Fusarium head blight and ear rot in small grain crops like wheat and maize. Besides, the invasion of the fungus on plants is often accompanied by the production of health-threatening mycotoxins—trichothecenes. Therefore, the control of *Fusarium graminearum* has always been a hot area of research. However, currently, the prevalence of these plant diseases around the world and the mycotoxin accumulation beyond safety limits indicate the necessity for more related research. In the last decades, researchers have sought to identify the key substances associated with the growth, propagation, and mycotoxin production of the fungus, such as carbon and nitrogen sources. Organic acids have always been considered antifungal agents due to their abundant H+ content. However, their comprehensive impact on fungi has been rarely investigated. This research focused on the effect of citric acid, a type of crop exudate and a common heavy-metal chelator, on the metabolism of *Fusarium graminearum*. The result was expected to provide a theoretical basis for further studies on plants-soil-fungi interactions and the reasonable utilization of citric acid in agriculture.

## INTRODUCTION

*Fusarium graminearum*, a globally notorious plant pathogenic fungus, causes significant yield loss and quality deterioration of crops every year, accompanied by the production of mycotoxins like trichothecenes, which threaten the sustainable development of agriculture and the health of animals and human beings (1). In China, 5%–10% of wheat yield loss is common due to Fusarium head blight, in which *F. graminearum* dominates as a causal agent (2). Across Europe between 2010 and 2019, 75 million tonnes of wheat (5% of food-grade wheat) exceeded the limit of 750 µg/kg deoxynivalenol (DON)—a type B trichothecene (3). Recently, to solve these challenging issues, strategies such as fungicide application (4), field management (5), and bacteria symbiosis (6) have been widely adopted. However, their effectiveness has been limited due to the lack of understanding of the fungus and its responses in the sophisticated ecological niche, indicating the need for further research.

As a soil-borne plant pathogen, *F. graminearum* interacts with plants and soil through physical contact and substance exchange (7). It has been reported that wheat cultivars with different resistance to Fusarium head blight possess varying rhizosphere *F. graminearum* abundance and soil chemical properties such as carboxylic acids and NH_4_^+^-N content, and rhizosphere *F. graminearum* abundance was positively correlated with the utilization of carboxylic acids (8). Among these acids, citric acid (CA), an important endogenous organic acid, was selected for further investigation. CA was excreted from plant roots into the soil in phosphorus-deficient and heavy metal-stressed conditions as a facilitator of nutrient intake or an enhancer of heavy metal tolerance (9). Besides, the finding of CA as an effective chelator of metal ions prompted its exterior utilization in the field of soil restoration (10–12). However, little is currently known about the influence of CA on *Fusarium* species except its role as a weak acid (13).

Considering the potentially complicated effects of citric acid on *F. graminearum* in terms of growth and metabolism, we conducted transcriptomic and proteomic analyses to investigate the comprehensive metabolic alterations in *F. graminearum* treated with gradient CA in this study. We also used other validation methods subsequently. We aimed to identify the overall changes in genes, enzymes, and metabolic pathways of the fungus treated with CA, which would provide insights into the underlying functions of CA on *F. graminearum*.

## MATERIALS AND METHODS

### Reagents and strain

*F. graminearum* PH-1 (NRRL 31084) was stored and activated in our laboratory using potato dextrose agar (PDA) medium purchased from Shanghai Bio-way Technology Co., Ltd. (Shanghai, China). Citric acid (CAS: 77-92-9, purity: 99%) was obtained from Sigma-Aldrich Co., Ltd. (Shanghai, China). Standard samples of deoxynivalenol (CAS: 51481-10-8, purity: 97%), 3-acetyldeoxynivalenol (3-ADON, CAS: 50722-38-8, purity: 98%), 15-acetyldeoxynivalenol (15-ADON, CAS: 88337–96-6, purity: 98%), and nivalenol (NIV, CAS: 23282-20-4, purity ≥99.0%) were bought from Shanghai Macklin Biochemical Co., Ltd. (Shanghai, China). All other chemical reagents were of analytical grade.

### Fungal culture and sample collection

The stored *F. graminearum* was activated on the PDA medium at 25°C for 5–6 days, and the fungal plugs on the margin of the medium were obtained for subsequent culture. The type B trichothecene biosynthesis inducing medium (TBI) was prepared according to a previous study with minor modifications (14): the medium consisted of 36 g sucrose, 2.0 g (NH_4_)_2_SO_4_, 1.0 g KH_2_PO_4_, 0.5 g MgSO_4_·7H_2_O, 0.5 g KCl, 10 mg FeSO_4_·7H_2_O, and 200 µL trace element solution per liter and was adjusted to pH 6.5 with NaOH. The trace element solution (100 mL) contained 5 g ZnSO_4_·7H_2_O, 0.25 g CuSO_4_·5H_2_O, 50 mg MnSO_4_·H_2_O, and 50 mg Na_2_MoO_4_·2H_2_O. For preparing the TBI solid medium, 15 g/L agar was added before autoclaving.

In an attempt to investigate the influence of CA on *F. graminearum*, TBI solid media containing 2.5, 5, 10, and 20 mM CA were prepared. Then, a 0.8 cm plug was inoculated in the center of each medium. After a 14-day culture in a 25°C, 45% RH incubator, the samples were collected, frozen in liquid nitrogen, and stored at −80°C for RNA and protein extraction. The untreated mycelium was used as a control.

### Type B trichothecene production and detection

*F. graminearum* conidia were prepared before the trichothecene induction assay. An activated *F. graminearum* plug was inoculated into a 75 mL 20/1,000 (wt*/*wt) mung bean broth and incubated in a 28°C shaker at 180 rpm for 4–5 days. After filtering the mycelium through a four-layer gauze, the broth was centrifuged (4°C, 4,000 rpm, and 5 min) to collect the deposit. The spores were washed and resuspended three times with sterile water. Before formal analysis, the spores stored at 4°C were adjusted with a hemocytometer.

The spores of *F. graminearum* were inoculated into a 20 mL TBI medium containing 5 or 10 mM CA to reach a final concentration of 1 × 10^3^ spores/mL. After a 7-day culture in a 25°C and 45% RH incubator according to the termination time of *Tri5* expression (15), the samples were centrifuged (4°C, 8,000 rpm, and 5 min). The liquid supernatant was stored at −20°C for mycotoxin assay, while the sediment was freeze-dried for biomass measurement. The untreated mycelium was used as a control.

Considering the efficiency of distinguishing DON, 3-ADON, 15-ADON, and NIV, high-performance liquid chromatography (HPLC, 1260 Infinity II, Agilent, USA) was adopted instead of enzyme-linked immunosorbent assay (16). The analysis was carried out at 219 nm using a diode array detector. The procedure was carried out following the previously reported method with modifications (17). For various trichothecenes in liquid culture, 20 µL of samples was injected with the following binary gradient conditions: ultra-pure water (A) and acetonitrile (B). The C18 column (Zorbax SB-C18, Agilent, USA; 4.6 mm × 250 mm, 5 µm) was maintained at 35°C, and the flow rate was 0.8 mL/min. The gradient started at 5% B for 5 min and increased linearly to 20% B, 30% B, and 40% B at 5, 10, and 15 min, respectively. Then, to remove the remnants, the mobile phase composition was changed to 100% B at 25 min and held for 10 min. After that, the gradient returned to 5% B for 10 min to allow reconditioning of the column. The retention time was 11.32 and 13.66 min for NIV and DON, respectively, while acetylated derivatives showed very close retention times: 18.84 and 19.24 min for 15-ADON and 3-ADON, respectively. The concentration of trichothecene was calculated from the peak area according to the corresponding standard curves (Fig. S1).

### pH determination

To monitor the environment during the growth of *F. graminearum*, the pH value of the TBI medium was determined using a long tube pH pen (AZ8692, AZOVTES, China) every 24 h.

### Transcriptomic analysis of *F. graminearum*

#### RNA extraction and Illumina sequencing

Total RNA was extracted from the *F. graminearum* samples using TRIzol reagent according to the instructions. The quality and quantity of RNA were determined by 5300 Bioanalyzer (Agilent, USA) and ND-2000 (Nanodrop Technologies, USA). High-quality RNA samples (OD_260/280_ = 1.8–2.2, OD_260/230_ ≥ 2.0, RQN ≥ 6.5, and 28S:18S ≥ 1.0) were used to construct the sequencing library.

Library construction was carried out according to the following procedures. First, mRNA was isolated using the polyA selection method with oligo (dT) beads and then fragmented using a fragmentation buffer. Second, double-stranded cDNA was synthesized using a Double-Stranded cDNA Synthesis Kit (Invitrogen, USA) with random hexamer primers. Then, the synthesized cDNA was subjected to end repair, phosphorylation, and adapter addition according to the library construction protocol. After quantification by Qubit 4.0, the sequencing library was prepared on the NovaSeq X Plus platform (PE150) using the NovaSeq Reagent Kit.

#### Transcriptomic data analysis

The raw paired-end reads were trimmed and quality controlled by fastp with default parameters. Then, clean reads were separately aligned to the reference genome with orientation mode using HISAT2 software. The mapped reads of each sample were assembled by StringTie. Gene expression levels were established using the transcripts per million reads method. Differential expression analysis was performed using DESeq2. Different genes with a fold change (FC) ≥ 2 and a false discovery rate-adjusted *P* < 0.05 were considered to be significantly different expressed genes (DEGs). In addition, functional enrichment analysis, including Gene Ontology (GO) and Kyoto Encyclopedia of Genes and Genomes (KEGG), was performed to identify which DEGs were significantly enriched in GO terms and metabolic pathways, and *P* < 0.05 was considered as the threshold.

### DIA proteomic analysis of *F. graminearum*

#### Protein extraction, quantification, digestion, and UPLC-MS/MS analysis

The protein was isolated and quantified by bicinchoninic acid assay. Tris(2-carboxyethyl)phosphine and iodoacetamide were used to break the disulfide bond and perform reductive alkylation of the protein to fully hydrolyze the protein. The proteins were then digested with trypsin, and the obtained peptides were quantified according to the instructions of the Colorimetric Peptide Assay Kit (Thermo Fisher Scientific, USA). The EASY-nLC 1200 system coupled with the Q-Exactive HF-X (Thermo Fisher Scientific, USA) was used for analysis. The data were recorded using Thermo Xcalibur 4.0 (Thermo Fisher Scientific, USA), and the acquisition mode was data-independent acquisition (DIA).

#### Proteomic data analysis

Proteome Discoverer and Spectronaut were used for data analysis and library construction. Different proteins with a FC ≥ 2 and a false discovery rate-adjusted *P* < 0.05 were considered to be significantly different expressed proteins (DEPs). The GO enrichment and KEGG enrichment were performed to facilitate the analysis of the biological functions of DEPs. The enriched terms and pathways were considered to be significant when *P* < 0.05.

### Real-time quantitative reverse transcriptional PCR validation

Real-time quantitative reverse transcription PCR (RT-qPCR) analysis was carried out for selected DEGs to validate the results of RNA-sequencing (RNA-seq). Total RNA was isolated from the cells of *F. graminearum* using TRIzol reagent, and cDNA was synthesized using a cDNA Synthesis Kit (Invitrogen, USA). Subsequently, RT-qPCR reactions were carried out to evaluate the transcription levels using the SYBR Green method (ABI 7500, Thermo Fisher Scientific, USA). The β-tubulin gene (FGSG_06611) was used as the housekeeping gene to normalize the expression levels. The corresponding primers of the detected genes are listed in Table S1.

### Parallel reaction monitoring-targeted proteomic validation

The parallel reaction monitoring (PRM) detection was implemented for selected proteins to validate the results of DIA proteomic analysis (UPLC-MS/MS). The proteins were hydrolyzed to gain the mixture of peptide segments that were loaded onto a C18 column (75 µm × 200 mm, 3 µm) in an Easy nLC 1200 system (Thermo Scientific, USA). Then, the PRM-based targeted MS of separated peptide fragmentations was performed through the Q-Exactive HF mass spectrometer (Thermo Scientific, USA) in positive ion mode. Finally, Skyline 3.5.0 was used for the processing of raw data. The relative amounts of targeted proteins among the CK, 5, and 10 mM groups were demonstrated according to the relative amounts of corresponding peptide segments after the correction of the internal standard.

### Statistical analysis and plotting

All the experiments were carried out with three biological repeats, and the values were expressed as mean ± standard error. Differences between samples were considered to be significant when *P* < 0.05. In addition to the software mentioned above, R (version 4.2.2), RStudio (version 1.2.5001), and GraphPad Prism 9 were used for data analysis and plotting.

## RESULTS

### Morphology of *F. graminearum* treated with CA gradient

**Fig 1:**
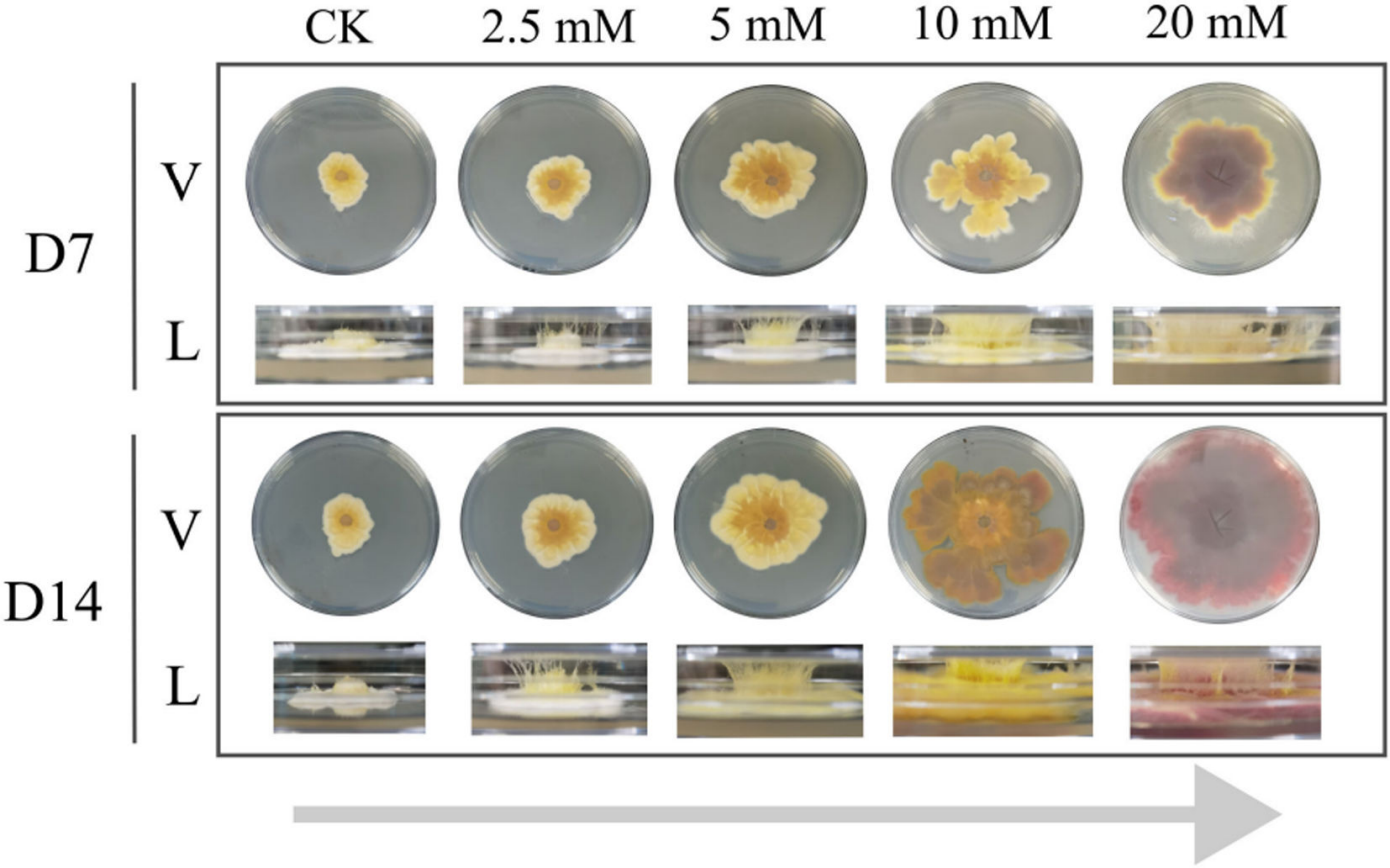
Morphology of *F. graminearum* grown on TBI solid medium. V, vertical view; L, lateral view.

The morphology of *F. graminearum* treated with 2.5, 5, 10, and 20 mM CA was observed on days 7 and 14 (Fig. 1). As the concentration increased, the morphology of the fungi changed. On one hand, the aerial and substrate mycelium were more vigorous. On the other hand, the color of the mycelium changed from yellow-golden to wine-red with increasing concentrations. In addition, the mycelium treated with 10 and 20 mM CA had a dramatic change from day 7 to day 14 compared with other groups. It has been reported that the color variation was caused by the biosynthesis and metabolism of a mixture of secondary metabolites such as yellow aurofusarin, red rubrofusarin, and orange carotenoid (18, 19). Thus, the results suggested that CA treatment ranging from 2.5 to 20 mM might accelerate the growth and alter pigment metabolism of *F. graminearum* in a dose-dependent manner, which piqued our interest in further investigation.

### General analysis of transcriptomes and proteomes

**Fig 2:**
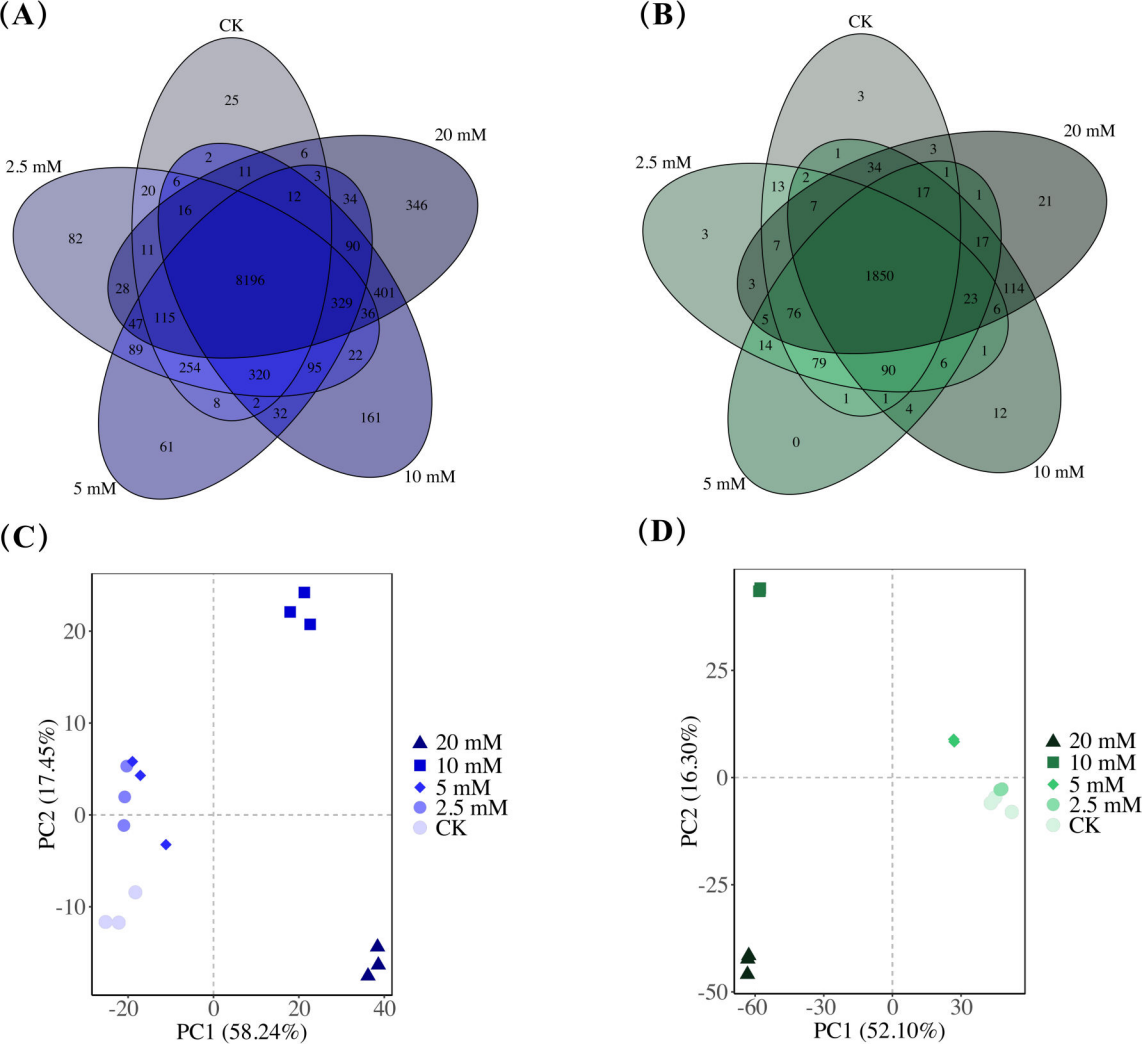
(**A**) Venn plots of mutual and different genes among the five groups. (**B**) Venn plots of mutual and different proteins among the five groups. (**C**) Principal component analysis of the transcriptomes among the five groups. (**D**) Principal component analysis of the proteomes among the five groups.

The transcriptome of *F. graminearum* treated with a CA gradient was determined by eukaryotic RNA-seq, while the proteomic analysis was performed using the DIA method. There were 25, 82, 61, 161, and 346 unique genes and 3, 3, 0, 12, and 21 unique proteins discovered in the five groups, respectively (Fig. 2A and B). Compared with the control, 574, 588, 2,444, and 3,023 upregulated and 735, 523, 2,357, and 3,023 downregulated DEGs were screened in the 2.5, 5, 10, and 20 mM groups (Fig. S2A), and 388, 345, 804, and 863 upregulated and 192, 519, 1,922, and 1,852 downregulated DEPs were screened in the 2.5, 5, 10, and 20 mM groups (Fig. S2B). Principal component analysis was performed to investigate the relationship among these five groups. As illustrated in Fig. 2C and D, CK, 2.5, and 5 mM groups were close in location, while these three groups, the 10 mM group, and the 20 mM group were far from each other, indicating that several key genes and corresponding proteins of *F. graminearum* might be activated or translated when treated with CA at a concentration higher than 5 mM.

### GO and KEGG enrichment analysis of transcriptomes and proteomes

**Fig 3:**
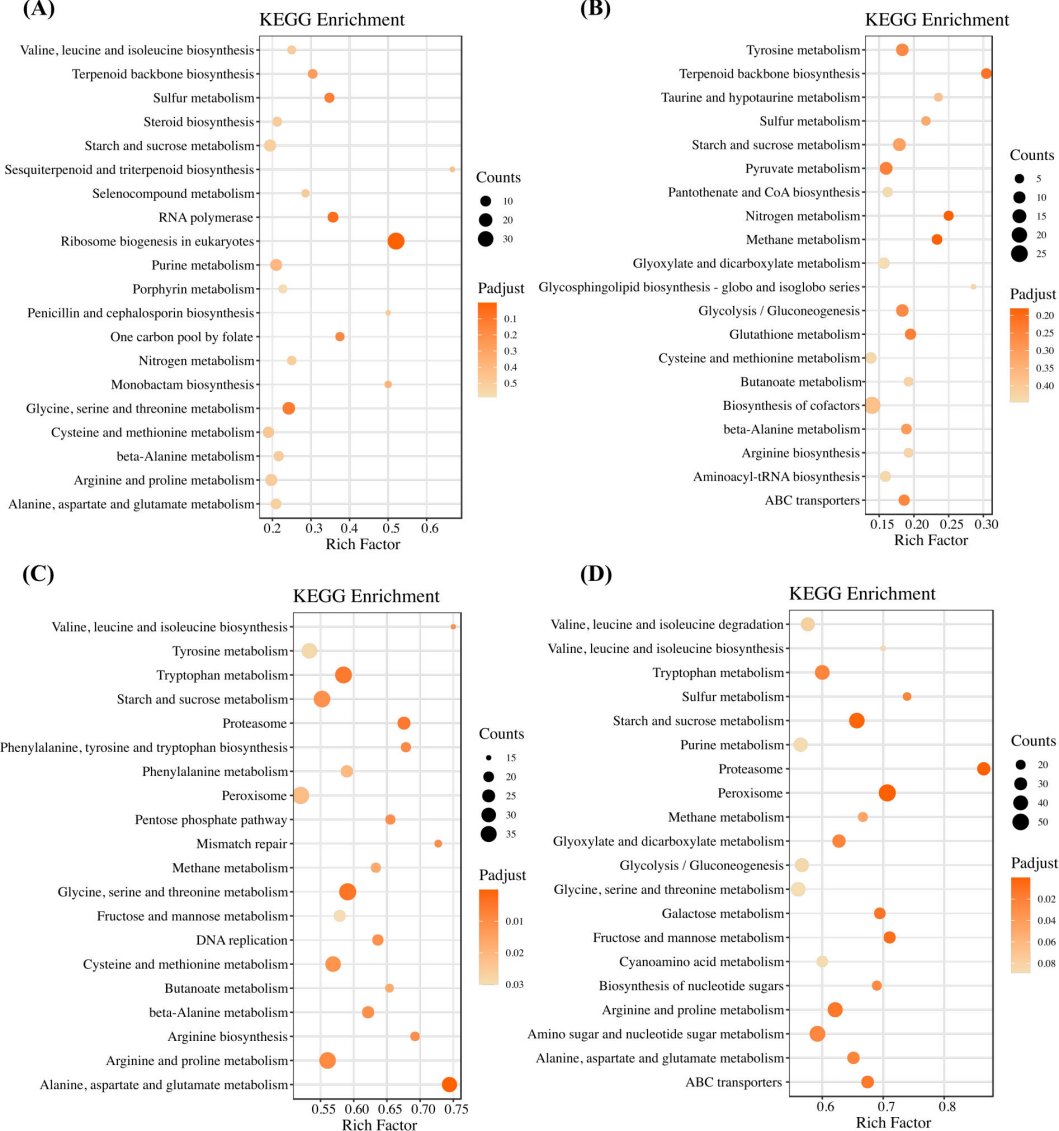
KEGG enrichment analyses of differentially expressed genes between (**A**) 2.5 mM and CK, (**B**) 5 mM and CK, (**C**) 10 mM and CK, and (**D**) 20 mM and CK.

Figure 3 and Fig. S3 demonstrate KEGG and GO enrichment analyses of DEGs in the comparisons of the 2.5, 5, 10, and 20 groups to the control, respectively. It was shown that several carbohydrate, amino acid, and organic acid metabolic processes were repeatedly found in the top 20 pathways and ontologies of four groups, such as glycolysis/gluconeogenesis, leucine and isoleucine biosynthesis, carboxylic acid metabolic process, suggesting that CA treatment may contribute to the growth of *F. graminearum* as a nutritional source. Interestingly, terpenoid backbone biosynthesis and sesquiterpenoid and triterpenoid biosynthesis were identified in the top 20 pathways of KEGG enrichment analyses of the 2.5 and 5 mM groups, indicating the possible influence of CA on the biosynthesis of trichothecenes, which belong to sesquiterpenoids.

**Fig 4:**
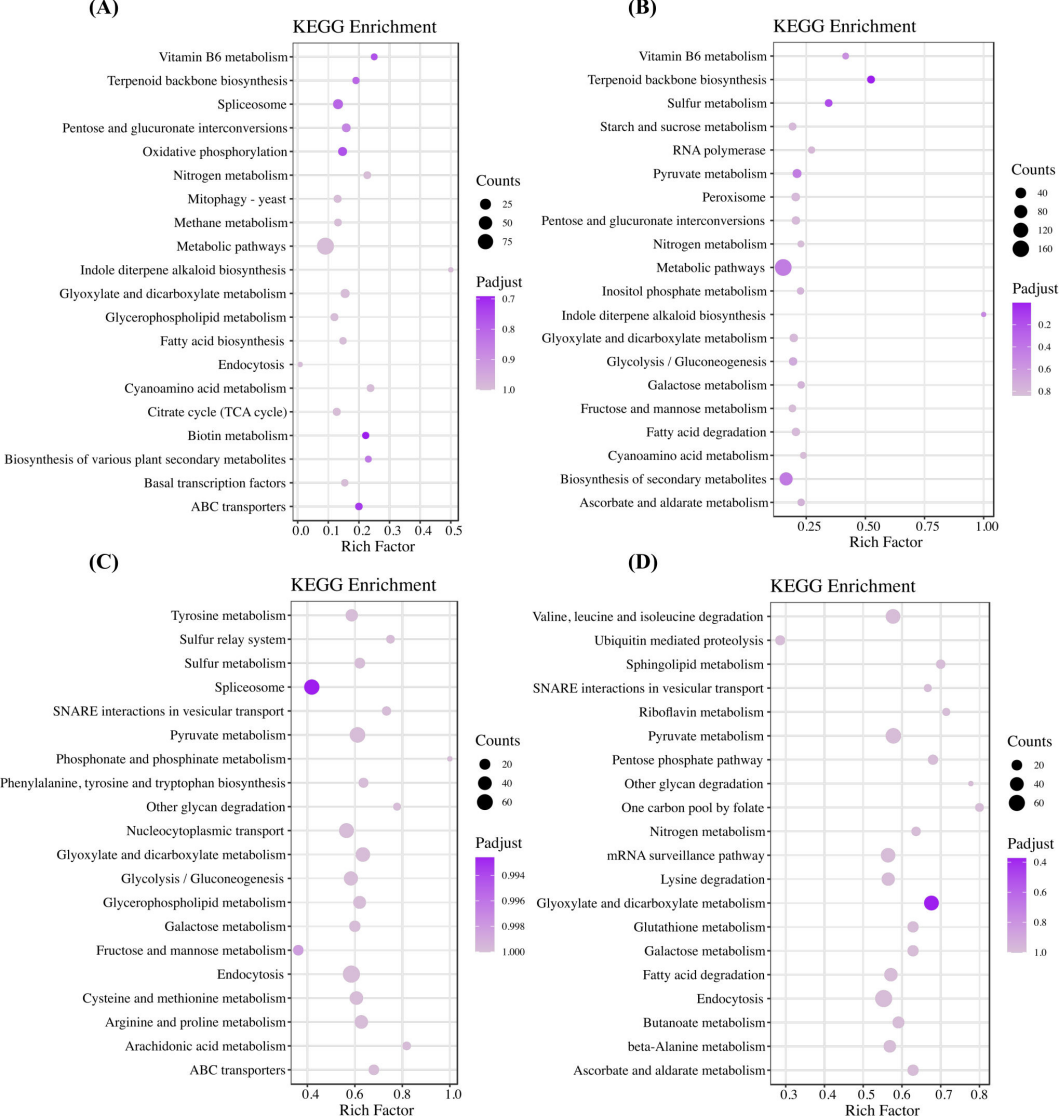
KEGG enrichment analyses of differentially expressed proteins between (**A**) 2.5 mM and CK, (**B**) 5 mM and CK, (**C**) 10 mM and CK, and (**D**) 20 mM and CK.

In the proteomic analyses shown in Fig. 4 and Fig. S4, there were also enriched ontologies and pathways related to the metabolisms of carbohydrates and nitrogen, such as cellular polysaccharide catabolic process, nitrogen metabolism, and intracellular protein transport. Of note, terpenoid backbone biosynthesis emerged in the top 20 pathways of KEGG enrichment analyses of the 2.5 and 5 mM groups, which was consistent with the transcriptomic results (Fig. 3).

### Association analysis of transcriptomes and proteomes

**Fig 5:**
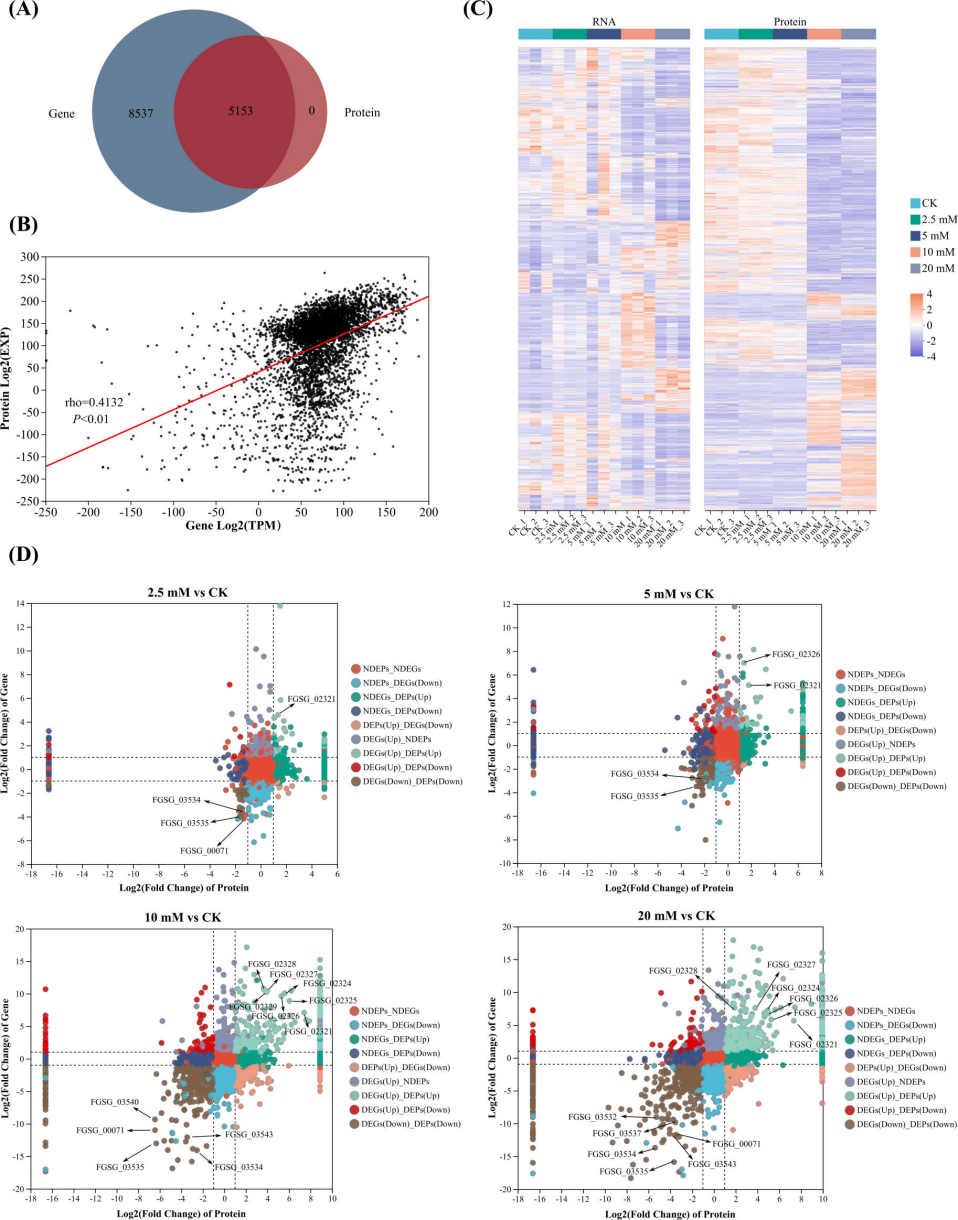
Association analysis of transcriptome and proteome. (**A**) Venn plot of genes and proteins detected. (**B**) Cluster analysis among five groups. (**C**) Correlation analysis between proteins and genes. (**D**) Differentially expressed genes and proteins. NDEPs/NDEGs: not differentially expressed genes or proteins.

As illustrated in Fig. 5A, all the corresponding genes of detected proteins could be found in the result of RNA-seq. The correlation coefficient of their expressions was 0.4132 (Fig. 5B). The cluster analyses of RNA and Protein among five groups are shown in Fig. 5C. It was obvious that the CK, 2.5, and 5 mM groups had similar RNA and protein expression patterns, while the 10 and 20 mM groups were alike. Importantly, many genes such as FGSG_03535, FGSG_03534, and FGSG_00071 and their corresponding proteins were downregulated in four CA treatment groups, while a lot of genes such as FGSG_02321, FGSG_02326, and FGSG_02324 and their corresponding proteins were upregulated in four treatment groups (Fig. 5D; Table 1). According to the previous studies, those downregulated genes and proteins played important roles in type B trichothecenes biosynthesis (20), and the upregulated ones were critical in pigment biosynthesis (19).

**TABLE 1:** Comparisons of gene and protein expressions in *F. graminearum* treated with citric acid at different concentrations^*a*^

Name	Gene ID	Protein ID	Log2 (fold change)							
			Transcriptomic				Proteomic			
			2.5 mM	5 mM	10 mM	20 mM	2.5 mM	5 mM	10 mM	20 mM
Growth										
*pckA*	FGSG_08601	XP_011320148.1	0.98^*b*^	1.88^*b*^	0.87^*b*^	5.47^*b*^	1.43^*b*^	1.48^*b*^	2.51^*b*^	3.05^*b*^
*glpk*	FGSG_03247	XP_011322504.1	2.48^*b*^	4.03^*b*^	5.63^*b*^	9.40^*b*^	1.32^*b*^	2.39^*b*^	6.54^*b*^	5.10^*b*^
*LAI12*	FGSG_02668	XP_011318618.1	0.60	0.64	5.95^*b*^	7.70^*b*^	0.62	1.43^*b*^	7.60^*b*^	7.44^*b*^
Zearalenone biosynthesis ABC transporter										
*Zra1*	FGSG_04580	XP_011321004.1	−2.52^*b*^	−4.83^*b*^	−12.67^*b*^	−10.23^*b*^	−1.12	−3.67	−4.49	−2.86
Pigment biosynthesis										
*PKS12*	FGSG_02324	XP_011318233.1	5.11^*b*^	5.90^*b*^	10.07^*b*^	7.24^*b*^	−0.46^*b*^	−0.52^*b*^	5.60^*b*^	4.96^*b*^
*aurF*	FGSG_02327	XP_011318236.1	6.99^*b*^	7.53^*b*^	10.40^*b*^	7.59^*b*^	−0.06	0.07	3.96^*b*^	3.80^*b*^
*aurJ*	FGSG_02326	XP_011318235.1	6.52^*b*^	6.70^*b*^	9.63^*b*^	6.76^*b*^	0.74	1.40^*b*^	5.30^*b*^	4.99^*b*^
*aurO*	FGSG_02321	XP_011318230.1	4.58^*b*^	5.10^*b*^	7.10^*b*^	5.64^*b*^	1.36^*b*^	1.80^*b*^	7.41^*b*^	7.34^*b*^
*aurS*	FGSG_02329	XP_011318238.1	7.01^*b*^	7.57^*b*^	10.21^*b*^	7.37^*b*^	0.75	1.05	3.89^*b*^	2.94^*b*^
*aurZ*	FGSG_02325	XP_011318234.1	5.00^*b*^	5.48^*b*^	8.90^*b*^	5.83^*b*^	0.69	0.86^*b*^	6.04^*b*^	5.27^*b*^
*gip1*	FGSG_02328	XP_011318237.1	7.12^*b*^	7.79^*b*^	10.58^*b*^	7.63^*b*^	−2.43^*b*^	−1.10^*b*^	3.68^*b*^	1.91^*b*^
*aurT*	FGSG_02322	XP_011318231.1	0.21	0.55	0.31	1.07	1.61^*b*^	1.75^*b*^	−0.20	−1.40^*b*^
Trichothecene biosynthesis										
*Tri6*	FGSG_03536	–^*c*^	−2.51^*b*^	−2.35^*b*^	−5.71^*b*^	−11.00^*b*^	–^*c*^	–^*c*^	–^*c*^	–^*c*^
*Tri10*	FGSG_03538	–^*c*^	−2.07^*b*^	−0.62	−2.85^*b*^	−6.53^*b*^	–^*c*^	–^*c*^	–^*c*^	–^*c*^
*Tri12*	FGSG_03541	XP_011322168.1	−3.70^*b*^	−2.80^*b*^	−9.97^*b*^	−12.40^*b*^	0.22	−1.99^*b*^	−16.61^*b*^	−16.61^*b*^
*Tri5*	FGSG_03537	XP_011322172.1	−3.48^*b*^	−2.85^*b*^	−10.15^*b*^	−9.85^*b*^	−0.94	−1.95	−3.51^*b*^	−3.53^*b*^
*Tri4*	FGSG_03535	XP_011322174.1	−3.96^*b*^	−3.49^*b*^	−13.03^*b*^	−15.88^*b*^	−1.64^*b*^	−2.68^*b*^	−6.33^*b*^	−3.65^*b*^
*Tri101*	FGSG_04692	XP_011323124.1	−0.50	−0.67	−4.89^*b*^	−4.26^*b*^	−0.09	−0.95^*b*^	−1.05^*b*^	−2.07^*b*^
	FGSG_07896	XP_011327728.1	−0.51	−0.02	−2.19^*b*^	−7.20^*b*^	−0.11^*b*^	−0.62^*b*^	1.33^*b*^	−3.72^*b*^
*Tri3*	FGSG_03534	XP_011322175.1	−3.71^*b*^	−2.84^*b*^	−14.08^*b*^	−13.70^*b*^	−1.42^*b*^	−2.24^*b*^	−2.54^*b*^	−5.40^*b*^
*Tri13*	FGSG_03542	–^*c*^	−3.97^*b*^	−3.22^*b*^	−10.32^*b*^	−9.94^*b*^	–^*c*^	–^*c*^	–^*c*^	–^*c*^
*Tri8*	FGSG_03532	XP_011322177.1	−1.35^*b*^	−0.65	−4.23^*b*^	−9.03^*b*^	−1.39^*b*^	−1.74^*b*^	−2.13^*b*^	−5.26^*b*^
*Tri7*	FGSG_03533	–^*c*^	−1.83^*b*^	−1.08	−4.79^*b*^	−6.38^*b*^	–^*c*^	–^*c*^	–^*c*^	–^*c*^
*Tri11*	FGSG_03540	XP_011322169.1	−3.99^*b*^	−3.48^*b*^	−9.34^*b*^	−14.06^*b*^	−0.36	−1.57	−6.45^*b*^	−1.79^*b*^
*Tri1*	FGSG_00071	XP_011315667.1	−4.17^*b*^	−3.47^*b*^	−10.99^*b*^	−11.74^*b*^	−1.33^*b*^	−2.46^*b*^	−6.44^*b*^	−3.44^*b*^
*Tri14*	FGSG_03543	XP_011322166.1	−4.33^*b*^	−3.46^*b*^	−12.08^*b*^	−11.79^*b*^	−0.73	−0.41	−3.45^*b*^	−3.59^*b*^
*PacC*	FGSG_12970	–^*c*^	−0.31	−0.22	2.09^*b*^	2.87^*b*^	–^*c*^	–^*c*^	–^*c*^	–^*c*^
Culmorin biosynthesis longiborneol synthase										
*CLM1*	FGSG_10397	XP_011319367.1	−4.21^*b*^	−7.06^*b*^	−17.03^*b*^	−17.62^*b*^	−1.79	−4.26	−16.61	−16.61

^*a*^ Comparisons of gene and protein expressions in *F. graminearum* treated with citric acid at different concentrations.

^*b*^ *P*＜0.05.

^*c*^ –, the corresponding protein was not detected in this study.

### Validation of inhibited type B trichothecene biosynthesis by CA treatment through RT-qPCR

**Fig 6:**
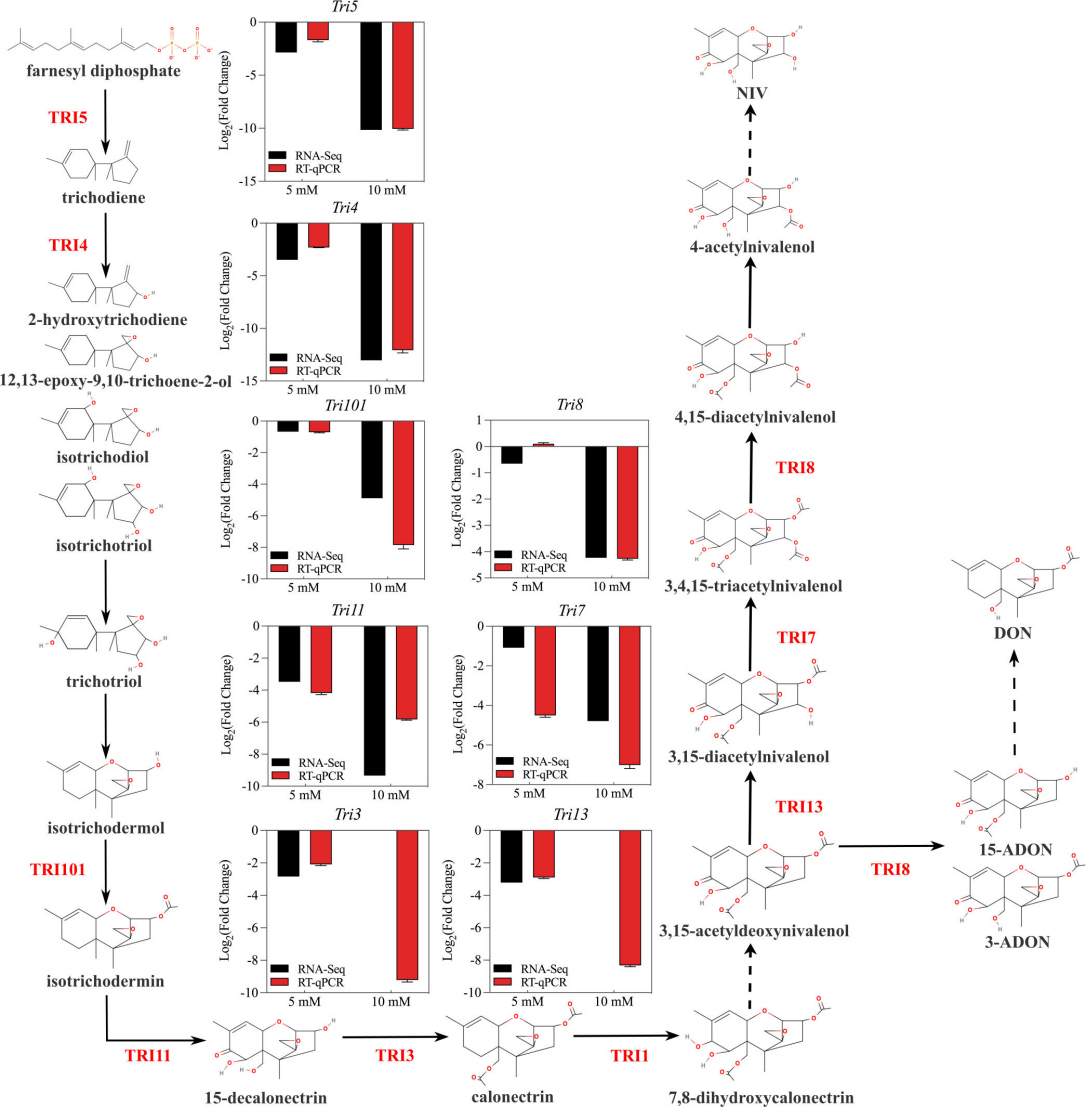
Schematic diagram of type B trichothecene biosynthesis, along with the RNA-seq and RT-qPCR results of several genes required for important steps.

**Fig 7:**
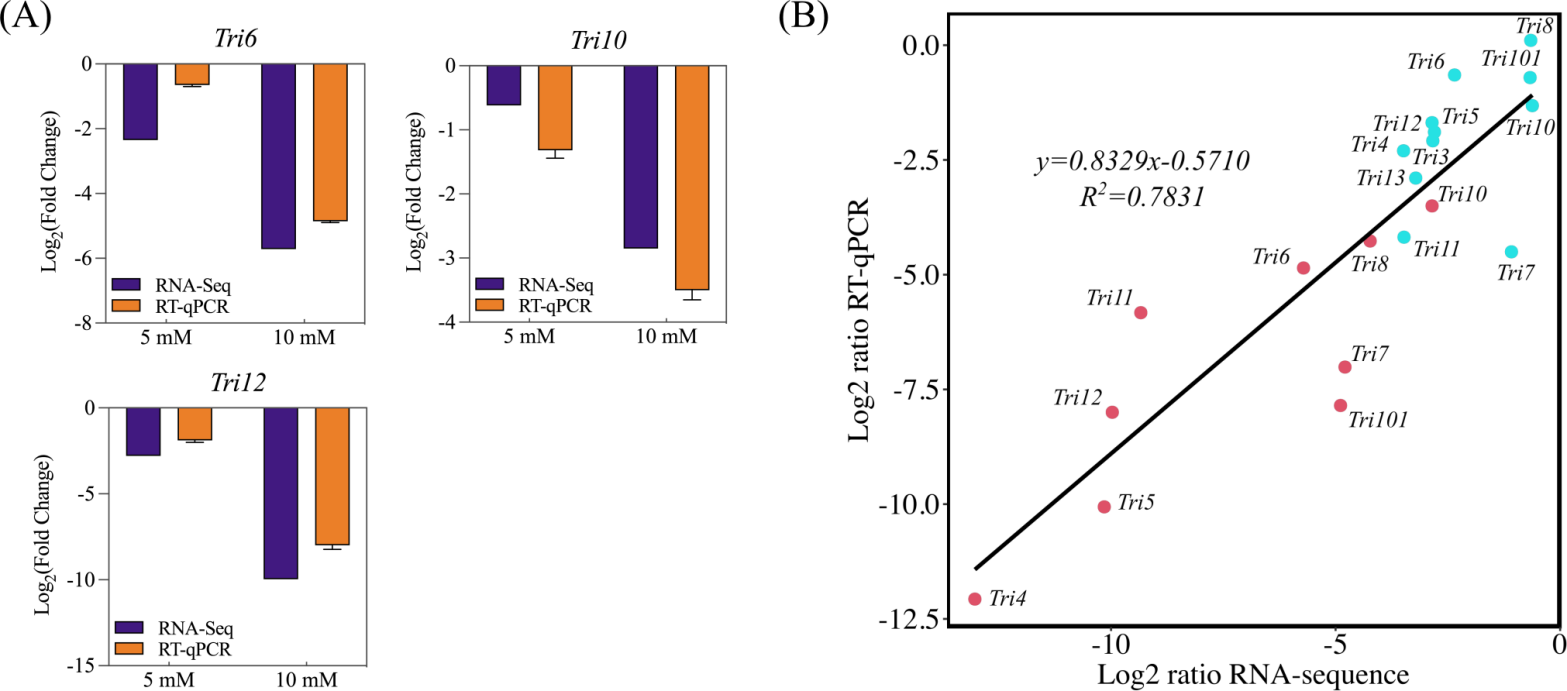
RT-qPCR validation. (**A**) RNA-seq and RT-qPCR results for *Tri6*, *Tri10*, and *Tri12* of the 5 and 10 mM groups. (**B**) Fit curve of RNA-seq and RT-qPCR for 11 *Tri* genes of the 5 and 10 mM groups.

Eleven *Tri* genes of the CK, 5, and 10 mM groups were selected for qRT-PCR analysis (Fig. 6 and 7A). The result of RT-qPCR correlated well with that of RNA-seq (Fig. 7B), verifying the influence of CA on type B trichothecene biosynthesis in *F. graminearum*. Besides, the production of four type B trichothecenes in the fungus was estimated through HPLC, in which 15A-DON and DON were detected (Fig. S5). As shown in Fig. S6A, DON production was reduced by CA in a dose-dependent manner. The production of 15A-DON was significantly decreased by 10 mM CA treatment; however, it was significantly increased by 5 mM CA treatment, which indicated the requirement for further investigation.

### PRM validation

**Fig 8:**
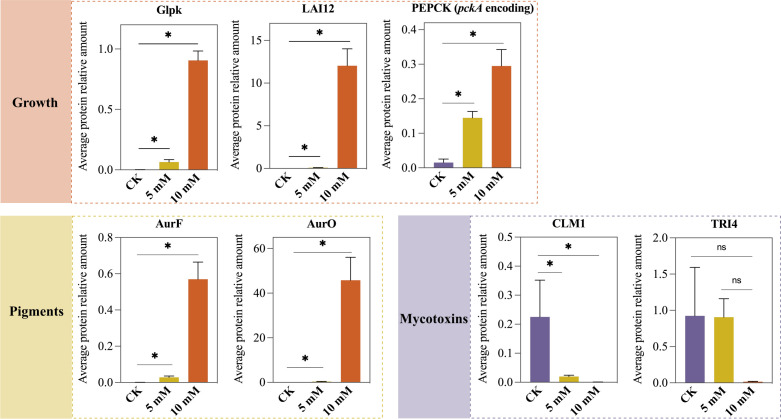
PRM validation of selected proteins related to growth, pigment biosynthesis, and mycotoxin biosynthesis. *P < 0.05.

Several proteins related to growth, pigment biosynthesis, and mycotoxin biosynthesis were selected for PRM detection. The average protein relative amounts of Glpk, LAI12, phosphoenolpyruvate carboxykinase (PEPCK), AurF, and AurO increased significantly when 5 or 10 mM CA was added, while the relative amounts of CLM1 and TRI4 decreased with increasing CA concentrations, which was generally in accordance with the DIA results (Fig. 8; Table 1).

## DISCUSSION

### Promoted growth

*pckA, glpk*, *LAI12,* and their corresponding enzymes were upregulated when *F. graminearum* was treated with a CA gradient, which may contribute to the growth of the fungus according to a previous study (Table 1). *pckA* encodes phosphoenolpyruvate carboxykinase, which is an essential enzyme for gluconeogenesis. It has been reported to positively influence the growth of *Staphylococcus aureus* and *Planctomyces limnophilus* in the absence of glucose (21, 22). Besides, *pckA*-deficient strains often performed differently in functions, such as virulence and hemagglutination (23, 24). In our study, sucrose, a trichothecene-inducing oligosaccharide containing an α-(1→2) (glucosyl/xylosyl)-fructosyl linkage (25), was the only carbon source in the medium. Thus, PEPCK may be one crucial enzyme that was upregulated by CA treatment in the glucose-free condition, leading to the growth of the fungus. *glpk* encodes an ATP-dependent phosphorylating enzyme called glycerol kinase, which is critical in carbohydrate metabolism, such as glycerol metabolism and dihydroxyacetone metabolism (26, 27), which may also influence the growth of *F. graminearum*. Linoleic acid isomerase (LAI12) is responsible for the transformation of linoleic acid to conjugated linoleic acid. Zhang et al. (28) reported that the disruption of *FgLAI12* in *F. graminearum* resulted in decreased mycelial growth. Therefore, the upregulated *LAI12* in our study might contribute to the promoted growth of the fungus.

### Altered pigments

Table 1 shows the list of enzymes regulating the biosynthesis of aurofusarin and rubrofusarin, which are considered to impact the surface color of *F. graminearum* the most (19). The biosynthesis of rubrofusarin could be completed by the polyketide synthase encoding gene *PKS12*, the dehydratase encoding gene *aurZ*, and the O-methyltransferase encoding gene *aurJ*, which has been realized in the engineered *Saccharomyces cerevisiae* (29). In order to convert rubrofusarin (red) into aurofusarin (yellow), laccase GIP1, monooxygenase AurF, oxidoreductase AurO, and AurS are required (30–32). Besides, AurT, a putative aurofusarin pump, also participated in the transformation of rubrofusarin to aurofusarin since the replacement of the *aurT* gene resulted in an increased rubro- to auro-fusarin ratio (33). In this study, most genes and proteins above were upregulated, indicating the possible enhanced biosynthesis of aurofusarin and rubrofusarin. Of note, AurT was downregulated in the 10 and 20 mM groups, which may lead to an increased rubrofusarin to aurofusarin ratio. These results are in accordance with the fungal appearances of the five groups shown in Fig. 1.

### Inhibited type B trichothecene biosynthesis

The biosynthetic gene clusters for type B trichothecenes in *Fusarium* have been partially elucidated in the last decades (Fig. 6). A core gene cluster containing 12 genes, a *Tri1-Tri16* cluster, and a single *Tri101* gene on different chromosomes are responsible for the synthesis and specific regulations (34). A trichodiene synthase TRI5 initiated type B trichothecenes biosynthesis through the catalysis of farnesyl diphosphate. Then, the multifunctional cytochrome P450 oxygenase TRI4 catalyzed successive reactions that led to the synthesis of isotrichoriol (35). After the spontaneous transformation from isotrichodermol to isotrichoriol, the C-3 acetyltransferase TRI101 took up the responsibility for converting isotrichodermol to isotrichodermin (36). Next, C-15 hydroxylase TRI11 and 15-O-acetyltransferase TRI3 completed the final steps to synthesize calonectrin (37, 38). Calonectrin is an important precursor for various trichothecenes produced by different *Fusarium* chemotypes with diverse activities of *Tri13*, *Tri7*, and *Tri8* (39–42). For the regulation of type B trichothecene biosynthesis, Tri6 and Tri10, two C_2_H_2_ zinc finger transcription factors, were validated to participate in the positive regulation of *Tri* genes (43–45). Besides, a putative major facilitator superfamily protein named TRI12, located in predicted motile vesicles, contributed to the export of trichothecenes and self-protection in *Fusarium* (46, 47). In our study, many *Tri* genes and their corresponding proteins were downregulated (Fig. 5D and 6 to 8), indicating that the CA gradient inhibited the production of type B trichothecenes from the aspects of biosynthesis, specific regulation, and export.

It was reported that the biosynthesis of type B trichothecenes was negatively regulated by the pH-responsive transcription factor PacC (48). The medium pH was monitored in our study, in which the decrease of pH in the medium was inhibited as the concentration of CA increased (Fig. S6B). Besides, *PacC* was significantly downregulated when treated with 10 and 20 mM CA (Table 1). Thus, it was proposed that the addition of CA enhanced the expression of *PacC*, realizing the adaptation of *F. graminearum* to the medium by repressing the acidification and inhibiting trichothecene biosynthesis through *Tri* genes. However, the corresponding protein was not detected in this investigation, indicating the need for further validation of this conjecture.

### Conclusion

Using multi-omics, this study investigated the influence of CA on *F. graminearum*, in which we found that CA contributed to the promoted growth, altered pigments, and inhibited type B trichothecene biosynthesis of the fungus. Interestingly, the downregulated genes and proteins related to growth and the upregulated ones associated with mycotoxin biosynthesis of *F. graminearum* were revealed in the meantime, suggesting that CA is a double-edged sword for the survival of the fungus, and the external application of CA on the soil requires more consideration in view of its positive effect on fungal growth. This study was expected to lay a theoretical foundation for the subsequent study about the regulation network of CA on *F. graminearum* and the proper utilization of CA in agricultural management.

## Data Availability

The transcriptomics data have been deposited to the SRA database with the data set identifier PRJNA1130548. The mass spectrometry proteomics data have been deposited to the ProteomeXchange Consortium via the iProX partner repository with the data set identifier PXD053628.

## SUPPLEMENTAL MATERIAL

Graphical abstract aem.01531-24-s0001.tiff Schematic of the study.

Supplemental material aem.01531-24-s0002.pdf Table S1; Fig. S1 to S6.

ASM does not own the copyrights to Supplemental Material that may be linked to, or accessed through, an article. The authors have granted ASM a non-exclusive, world-wide license to publish the Supplemental Material files. Please contact the corresponding author directly for reuse.
