# Supplementary figures and images for: Citric acid impairs type B trichothecene biosynthesis of Fusarium graminearum but enhances its growth and pigment biosynthesis: transcriptomic and proteomic analyses
Source: Appl Environ Microbiol. 2025 May 14;91(6):e01531-24. doi: 10.1128/aem.01531-24 (PMC12175541; doi:10.1128/aem.01531-24)

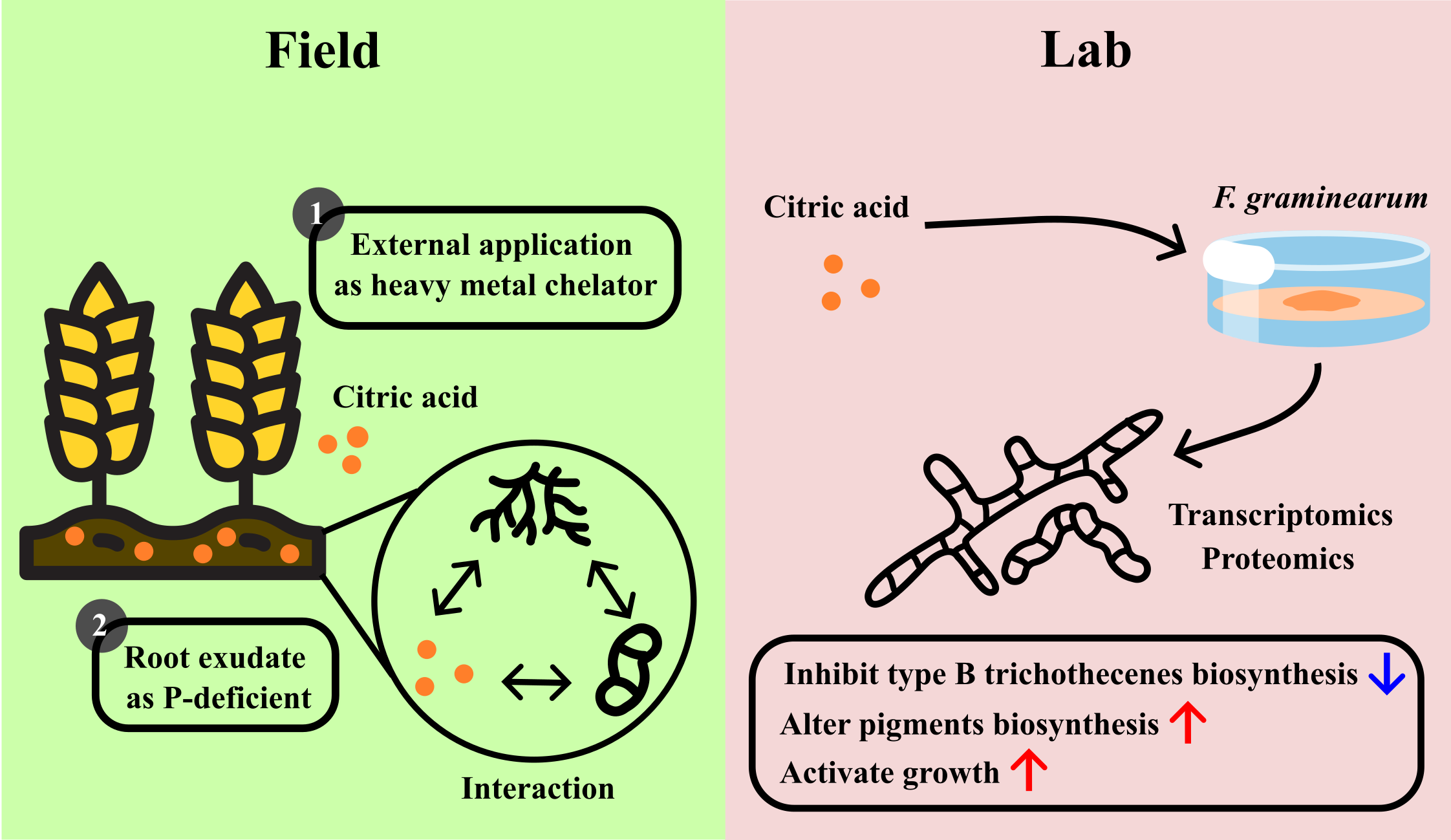

Supplement: Graphical abstract — Schematic of the study. [file aem.01531-24-s0001.tiff]
