# Supplementary material for: Citric acid impairs type B trichothecene biosynthesis of Fusarium graminearum but enhances its growth and pigment biosynthesis: transcriptomic and proteomic analyses
Source: Appl Environ Microbiol. 2025 May 14;91(6):e01531-24. doi: 10.1128/aem.01531-24 (PMC12175541; doi:10.1128/aem.01531-24)
Supplement: Supplemental material — Table S1; Fig. S1 to S6. [file aem.01531-24-s0002.pdf]

Supplementary information

Table S1. The corresponding primers of genes used for RT-qPCR validation

Gene	Nucleotide sequence of primers (5'-3')	
	Forward primer	Reverse primer
β-tubulin (housekeeping gene)	CAGGTCCACCGTCTCCAAGG	CACGCTGCTGGTCTGAAGTTC
Tri5	TCAGGATACAGAGGACGCCAAG	TCGCCTTCGGTTACTCCACTAG
Tri4	ATGGCAAGCACAACGACTATCTC	GTCCTGAATCTCATCTCGCATCTG
Tri101	TTATACTGGTGCGAGGCGATTG	TGTTGGTTAAGCGGCGGATATAC
Tri3	GTGCCGACGACTCCATTGC	GCCACTTGCGACCATCTACTG
Tri13	CATCCTCACTCCAGCACTTGTC	GCTCCAATGTCGTGGTCATCC
Tri8	CGCTGTTCTATCGCTTGGTCTG	ACGCACCGCCAGAGTAGC
Tri7	GCCGCCGCTGTTTGGAAG	ATGGAACTACTGATGGTTGTGAGAG
Tri6	TCGCTACTCAGAATGCCCTCAG	CATTGTTGTCCTTCCTTGTCTTGC
Tri10	GCTGTTGACTATACTGACCTCTGC	CTCTTCCATACCATCGCCTGTTC
Tri12	TTGTTCCATCTCATCAGGCATATCC	TGGCTGCGTTCTTGTTATCTCG
Tri11	ACGGTGGGAGACCTGACG	AGCCAACGCTCTGGAATGA

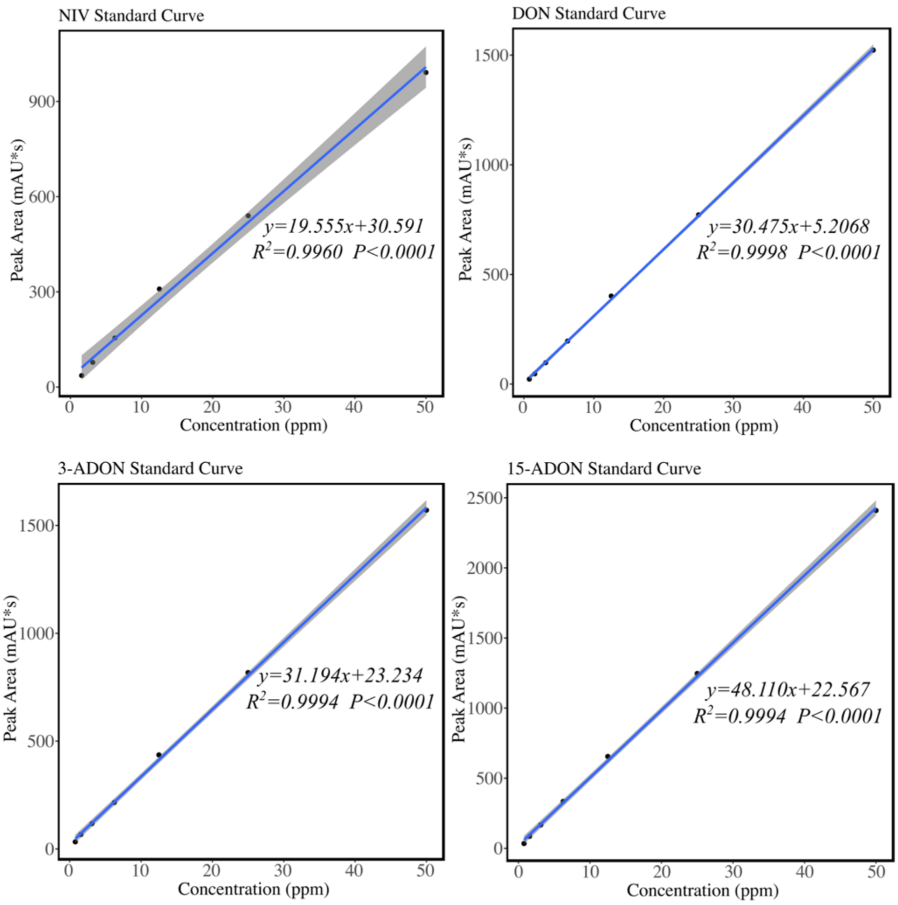

Fig. S1. The HPLC standard curves for NIV, DON, 3-ADON, and 15-ADON.

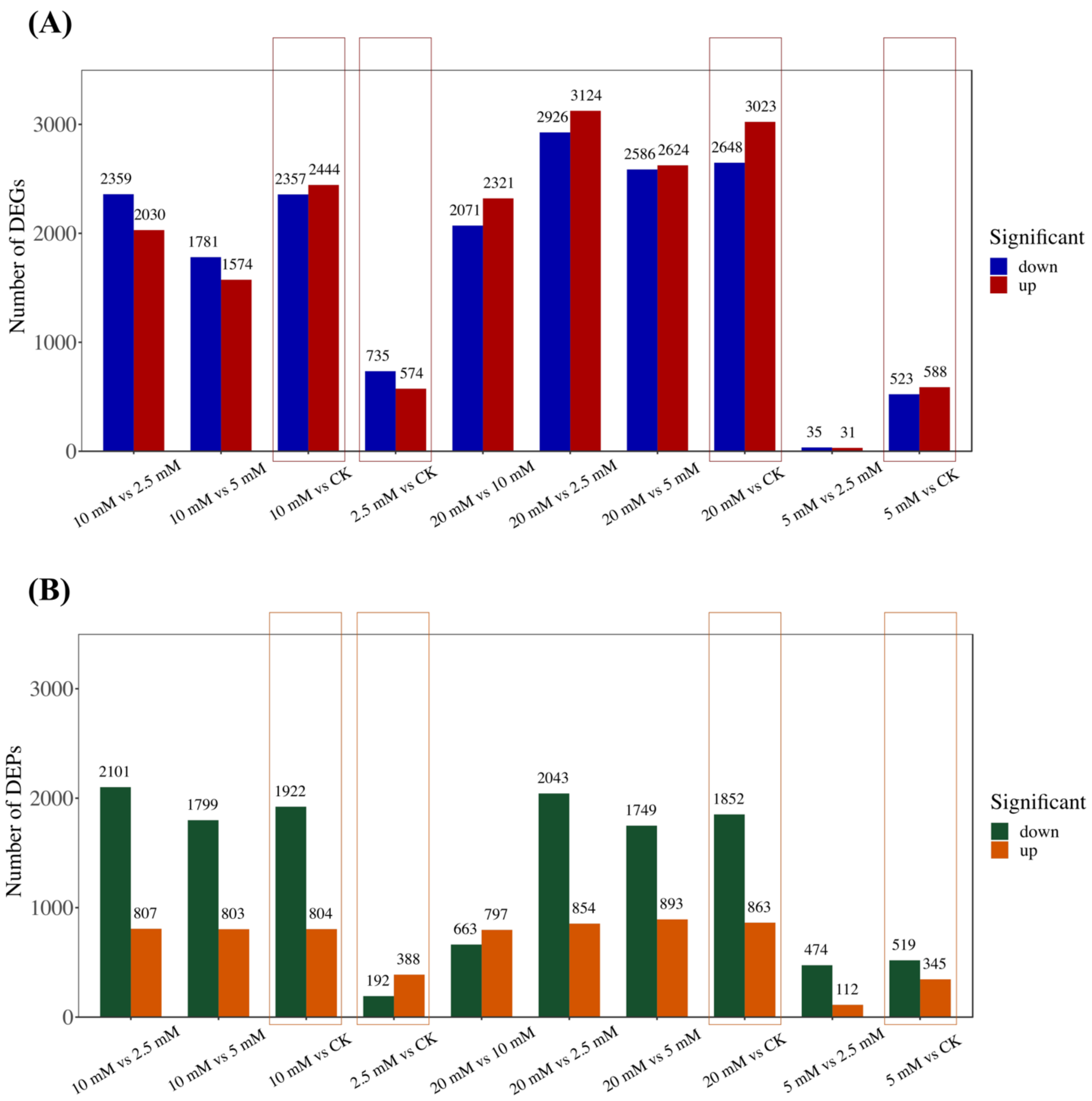

Fig. S2. (A) Differentially expressed genes among CK, 2.5 mM, 5 mM, 10 mM, and 20 mM groups. (B) Differentially expressed proteins among CK, 2.5, 2.5 mM, 5mM, 10 mM, and 20 mM groups.

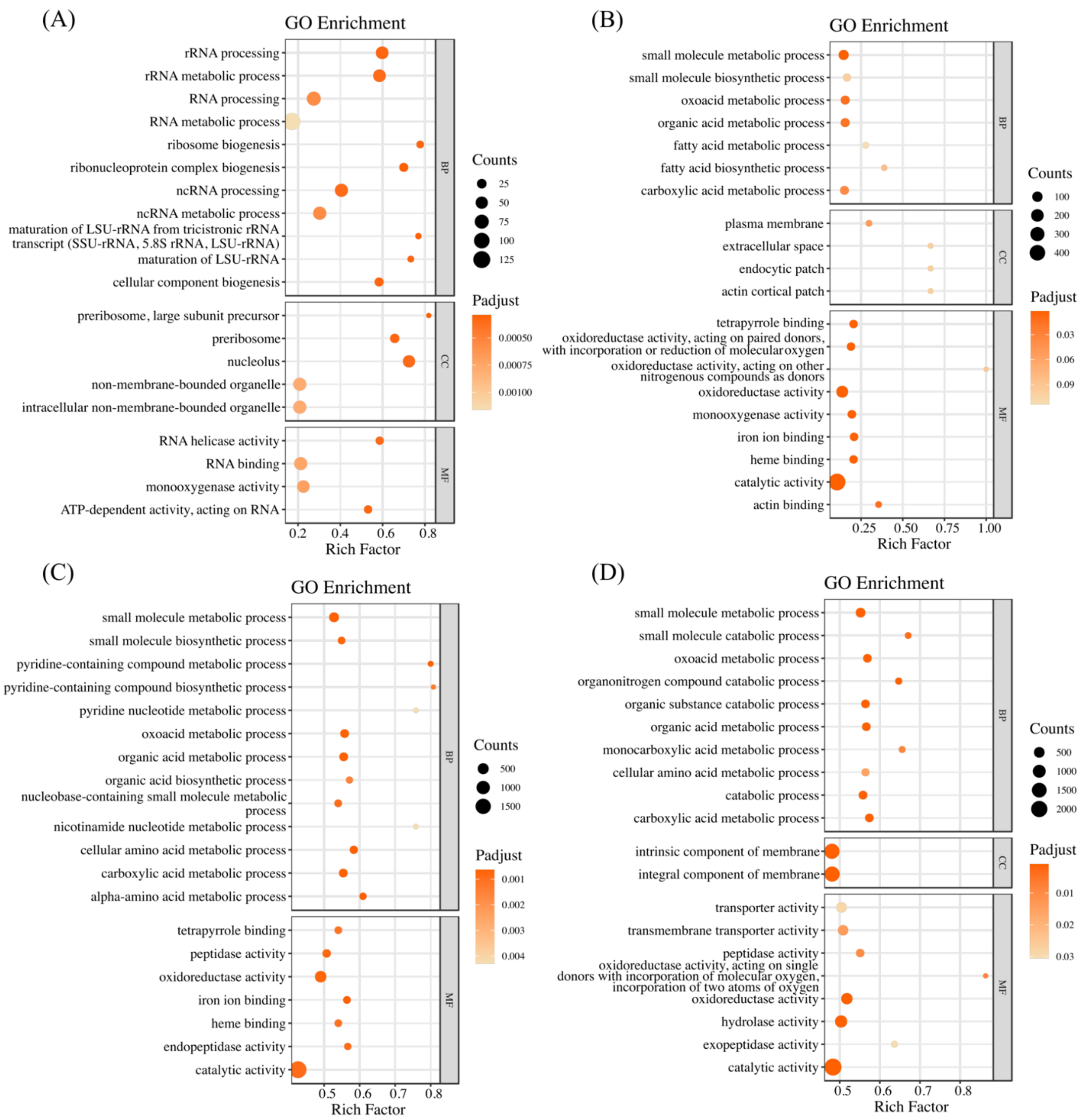

Fig. S3. The GO analysis of differentially expressed genes between (A) 2.5 mM and CK; (B) 5 mM and CK; (C) 10 mM and CK; (D) 20 mM and CK. BP: biological process, CC: cellular component, MF: molecular function.

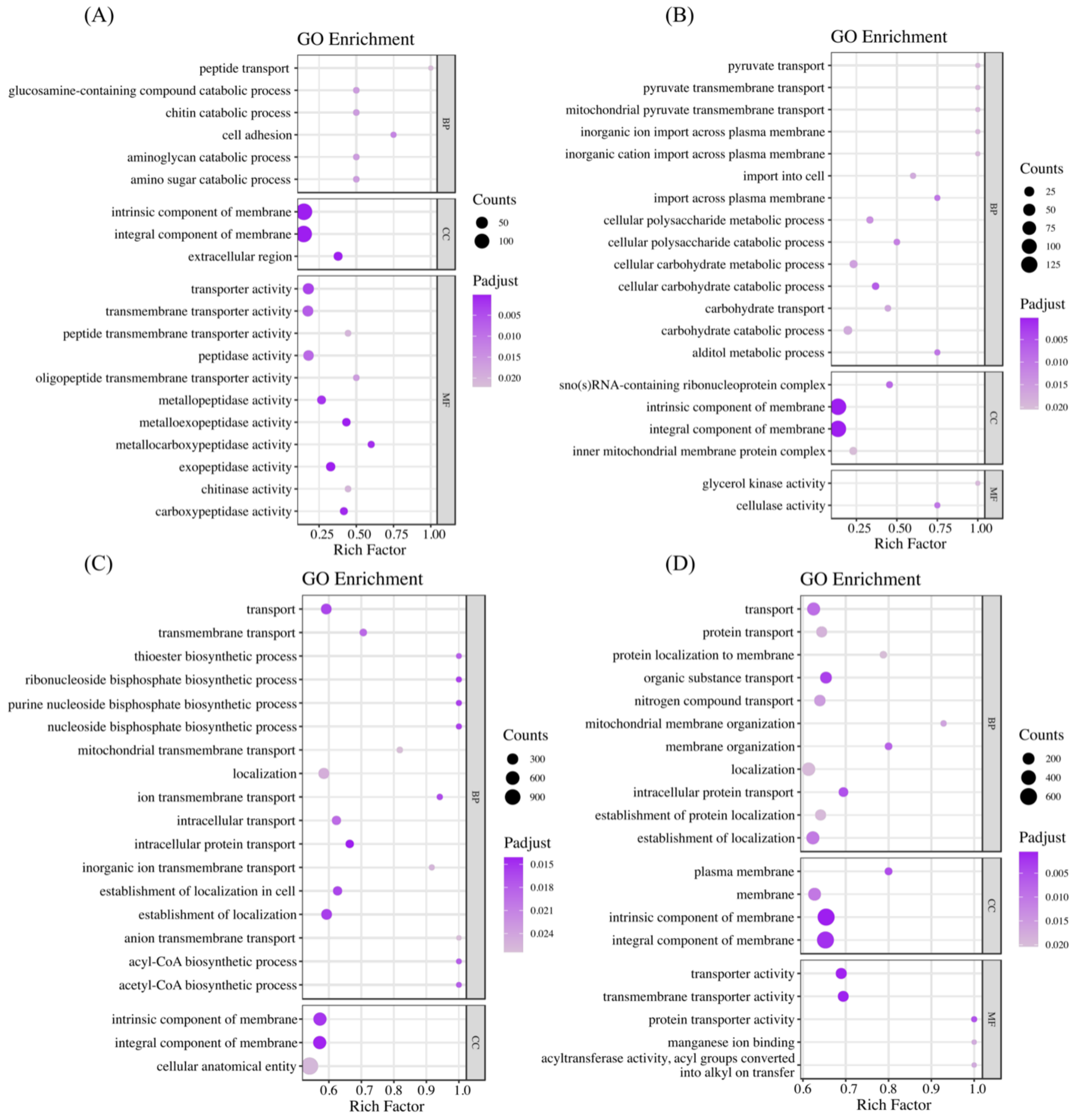

Fig. S4. The GO analysis of differentially expressed proteins between (A) 2.5 mM and CK; (B) 5 mM and CK; (C) 10 mM and CK; (D) 20 mM and CK.

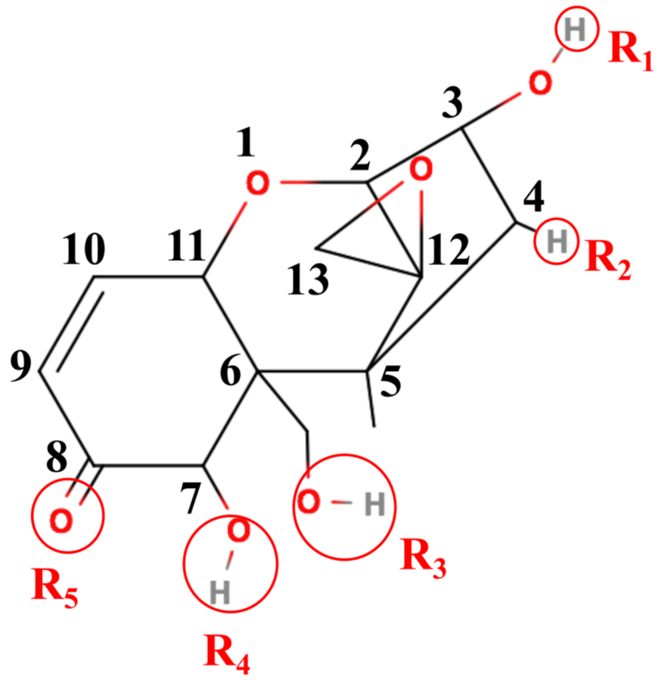

	R_1	R_2	R_3	R_4	R_5
DON	-OH	-H	-OH	-OH	=O
3-ADON	-OAc	-H	-OH	-OH	=O
15-ADON	-OH	-H	-OAc	-OH	=O
NIV	-OH	-OH	-OH	-OH	=O

Fig. S5. The structures of four type B trichothecenes.

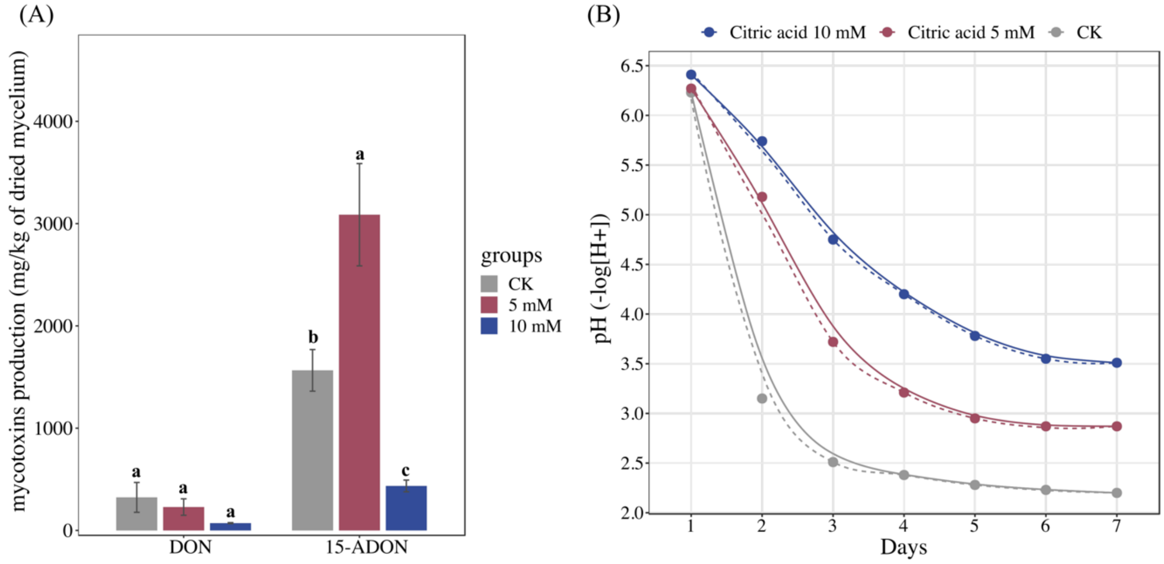

Fig. S6. (A) The mycotoxin productions of *F. graminearum* in the TBI liquid medium with 5 mM and 10 mM CA. The samples were collected on the 7th day. (B) The pH variation of the TBI liquid medium with 5 mM and 10 mM CA.
